# Delphinidin-3-glucoside suppresses breast carcinogenesis by inactivating the Akt/HOTAIR signaling pathway

**DOI:** 10.1186/s12885-016-2465-0

**Published:** 2016-07-07

**Authors:** Xiaohong Yang, En Luo, Xin Liu, Bin Han, Xiaoping Yu, Xiaoli Peng

**Affiliations:** Department of Public Health, Chengdu Medical College, Chengdu, China; Department of General Surgery, The Fifth People’s Hospital of Chengdu, Chengdu, China

**Keywords:** Breast cancer, Carcinogenesis, Anthocyanin, HOTAIR, lncRNAs

## Abstract

**Background:**

The long non-coding RNA (lncRNA) HOX transcript antisense RNA (HOTAIR) plays a crucial role in cancer progression, which is regulated by the interferon regulatory factor-1 (IRF1) and up-streaming Akt activation. The present study evaluated the chemopreventive effects of delphinidin-3-glucoside (Dp), a major anthocyanin present in pigmented fruits and vegetables, on breast carcinogenesis, and investigate the role of the Akt/HOTAIR signaling pathway.

**Methods:**

Human breast epithelial cells MCF10A were treated with carcinogens (NNK and B[a]P) or co-treated with carcinogens plus Dp for 30 days. Then, the cancer-associated properties of the treated cells were evaluated to assess the carcinogenesis and the effects of Dp. HOTAIR levels were detected by qRT-PCR. The proteins expression was measured by western blots, immunofluorescence and immunohistochemistry. Xenografted tumors were made by implanting breast cancer cells MDA-MB-231-Luc-GFP in athymic mice. ChIP-qPCR analysis was used to detect the IRF1 binding to the HOTAIR promoter.

**Results:**

Carcinogens treatment induces apparent carcinogenic transformation in MCF10A cells including reduced dependence on growth factors, anchorage-independent cell growth and aberrant wound-healing ability, which is effectively suppressed by Dp co-treatment. The level of HOTAIR significantly increases in a time-dependent manner during chronic breast carcinogenesis. Dp treatment down-regulates HOTAIR expression in breast carcinogenesis and breast cancer cells. Furthermore, Dp administration inhibits the growth of xenografted breast tumors in athymic mice, and decreases HOTAIR in vivo. Further studies showed that Dp represses Akt activation, promotes IRF1 expression and increases IRF1 binding to the HOTAIR promoter. Silence of IRF1 expression via transfecting cells with IRF1 siRNAs significantly reduced the effects of Dp on HOTAIR, resulting in decreased cytotoxic effects of Dp on breast cancer cells.

**Conclusions:**

These data suggest the effective chemopreventive effect of Dp on breast carcinogenesis, in which down-regulation of HOTAIR plays a critical role.

## Background

Breast cancer (BC) is a common and leading cause of cancer deaths among women worldwide [1]. Notably, BC is a serious health threat to women in Western countries. For a woman in the USA, the risk of a BC diagnosis during her lifetime is 12.5 %. More than 230,000 new cases of invasive BC are diagnosed annually in the USA, and approximately 40,000 women are expected to die from this common cancer [2, 3]. Despite advances in screening, diagnosis, and therapy, BC continues to pose an enormous global healthcare problem. Therefore, the identification of effective chemopreventive agents for BC, particularly dietary components, is important because it may lead to potential preventative therapies [4, 5].

High fruit and vegetable consumption is associated with extensive beneficial health effects, which are partially due to the bioactivities of phytochemicals in plant-based foods. Anthocyanins, a subclass of flavonoids, are a group of natural polyphenol compounds that are widely found in berries, red grapes, purple potatoes, red cabbages, and many other pigmented fruits and vegetables. Over 400 anthocyanins have been identified in nature, and studies revealed that these polyphenol compounds have a number of bioactivities, including anti-oxidant, anti-inflammation, anti-atherosclerosis, and anti-cancer properties [6–8].

Long non-coding RNAs (lncRNA), which are generally defined as RNA genes longer than 200 nucleotides that are not protein coding and represent a new family of regulatory RNAs that exert their function via diverse mechanisms [9–11]. Many studies revealed that lncRNAs play crucial roles in carcinogenesis and cancer progression. The HOX transcript antisense RNA (HOTAIR) is one of the first identified lncRNA and is transcribed from the antisense strand of the HOXC locus. This regulatory RNA is over-expressed in breast cancer, lung cancer and several other cancers. It acts as an oncogene by promoting cancer cell viability, growth and metastasis [12–14]. HOTAIR is regulated by the interferon regulatory factor-1 (IRF1) protein, which binds the HOTAIR promoter and inhibits its activity, thereby decreasing HOTAIR expression. Akt activation decreases IRF1 expression and consequently elevates the level of HOTAIR [15, 16]. In the present study, we evaluated the chemopreventive effects of delphinidin-3-glucoside (Dp), a major anthocyanin present in pigmented fruits and vegetables, on breast carcinogenesis and further investigated the role of the Akt/HOTAIR signaling pathway in the anti-cancer mechanism of Dp on breast cancer.

## Methods

### Chemicals and reagents

Dp was purchased from Mansite Bio-technology Co (Chengdu, China); DMEM/F12 medium and FBS were purchased from HyClone (Beijing, China); Trizol reagent, horse serum, gentamicin, insulin, Lipofectamine 2000, Opti-Mem were purchased from Invitrogen (Carlsbad, CA, USA); Epidermal growth factor (EFG) was purchased from PeproTech Inc (Rocky Hill, USA); PathScan Phospho-Akt ELISA assay kit and all antibodies were purchased from Cell Signaling Technology (Danvers, MA, USA). 3-(4,5-dimethylthiazol-2-yl)-2,5-diphenyl tetrazolium bromide (MTT), 4-(methylnitrosamino)-1-(3-pyridyl)-1-butanone (NNK), benzo[a]pyrene (B[a]P), cholera enterotoxin, hydrocortisol, dimethylsulfoxide (DMSO), phosphate buffered saline (PBS) and other chemicals were purchased from Sigma-Aldrich (St. Louis, MO, USA). All cell lines were purchased from Institute of Biochemistry and Cell Biology, Chinese Academy of Sciences (Shanghai, China)

### Chronic cellular breast carcinogenesis

Human breast epithelial cells MCF10A was maintained in complete medium (CM) (DMEM/F12 medium supplemented with mitogenic additives including 100 ng/ml cholera enterotoxin, 10 μg/ml insulin, 0.5 μg/ml hydrocortisol, 20 ng/ml EFG, and 5 % horse serum.) in a humidified atmosphere of 5 % CO_2_/95 % air at 37 °C. The cellular breast carcinogenesis model was processed as reported previously [17, 18]. Briefly, MCF10A cells were treated with NNK and B[a]P (each at 100 pmol/L) along with different concentrations of Dp; cultures were subcultured every 3 d. Cancer-associated properties of treated cells were evaluated by following assays. Reduced dependence on growth factors (RDGF) assay: 3 × 10^3^ cells were seeded in low-mitogen medium (LM), in which the contained total serum and mitogenic additives reduced to 2 % of the concentration formulated in CM; Growing colonies that reached 0.5 mm diameter in 10 d were counted. Anchorage-independent cell growth (AIG) assay: The base layer consisted of 2 % low-melting agarose in CM medium. Then, soft agar consisting of 0.4 % low-melting agarose in a mixture (1:1) of CM medium with 3-d conditioned medium prepared from MCF10A cultures was mixed with 5 × 10^3^ cells and plated on top of the base layer in 60-mm diameter culture dishes; Growing colonies that reached 0.1 mm diameter in 20 d were counted. Scratch/wound healing assay: Cells were seeded on 6-cm dishes and grown to confluence. The cell monolayer was scraped with a sterile cell scraper to create a cell-free zone to produce wounded cultures; the wound healing areas by cells were examined at 12 h and 24 h by subtracting the area not healed from total area of initial wound.

### Cell viability assay

Cells were planted in 24-well plates at a density of 10^5^ cells/well overnight, and treated with Dp. At the end of the treatment, 40 μl of MTT (5 mg/ml) were added to each well and the cells were cultured for another 4 h. The formazan crystals were dissolved in DMSO, and the absorbance was measured at 490 nm on a Bio-Rad automatic EIA analyzer.

### qRT-PCR analysis of HOTAIR

Total RNA was extracted with the Trizol reagent, and reverse transcription was performed using oligo (dT) 20 as primer and M-MLV reverse transcriptase (Promega, USA) at 42 °C for 30 min. HOTAIR levels were quantified using LightCycler 480 Probes Master kit (Roche Applied Science) following the manufacturer’s protocol with the following specific HOTAIR primers (forward 5′-ACGGAACCCATGGACTCATA-3′, reverse 5′-TTGGGGAAGCATTTTCTGAC-3′).. All samples were read in triplicate, and values were normalized to β-actin (forward 5′- TGACAGGATGCAGAAGGAGA-3′, reverse 5′-TAGAGCCACCAATCCACACA-3′).

### Western blot analysis

Cell lysates were prepared using RIPA buffer (25 mM Tris–HCl, pH 7.6, 150 mM NaCl, 1 % NP40, 1 % sodium deoxycholate, 0.1 % sodium dodecyl sulfate (SDS)) supplemented with protease and phosphatase inhibitors. Equal amounts of cellular proteins were resolved by electrophoresis in 10 % or 12 % SDS-polyacrylamide gels for Western immunoblotting with specific antibodies. Antigen-antibody complexes on filters were detected by chemiluminescence.

### Xenografted tumors in athymic mice

Female BALB/c nude mice were implanted with MDA-MB-231-Luc-GFP cells at a density of 2 × 10^6^ cells/ml s.c. into the right axilla, and randomly divided into the control and Dp administration groups. 72 h after implantation, the mice were i.g. orally fed Dp (40 mg/kg/day) or vehicle alone (normal saline). Mice under anaesthesia were injected i.p. with 15 mg/ml of D- luciferin (Sinochrome, shanghai, China) in DPBS, and images were recorded by the IVIS Imaging System (IVIS Spectrum, USA) after the injection. Mice were sacrificed at day 28 post-implantation and the weight of tumors was examined.

### Immunohistochemistry

The tissue sections (4-μm-thick) were placed onto treated slides, heat-fixed, deparaffinized, rehydrated, subjected to antigen retrieval for immunohistochemistry, Sections were stained for H&E for morphological study. After washing with PBS, the slides were blocked with 2 % serum for 0.5 h and then incubated with antibodies at 4 °C overnight. The secondary biotinylated antibody was then applied, and the signal was developed using a modified avidin-biotin complex immunoperoxidase staining procedure. Counterstaining was performed with Trypan blue or Harris hematoxylin. Immunostaining density was quantified using Image J analysis.

### p-Akt ELISA

The activity of p-Akt (S473) were measured with a PathScan Phospho-Akt (S473) ELISA assay kit. Briefly, select 10^7^ cells, washing 2 times with tris-buffered saline (TBS), and prepare cell lysates by adding 1 ml lysis buffer (50 mM Tris HCl, pH 7.4, 100 mM NaCl, 50 mM β-glycerophosphate, 10 % glycerol (w/v), 1 % Tween®-20 detergent (w/v), 1 mM EDTA, 20 nM microcystin-LR, 25 mM NaF, and a cocktail of protease inhibitors). p-Akt (S473) proteins in cell lysate were captured by the corresponding antibody that was coated in the microplate. After adding the horseradish peroxidase-linked secondary antibody and chemiluminescent substrate, the magnitude light emission, which is proportional to the quantity of p-Akt, was measured.

### Immunofluorescence

Cells were cultured and treated in 6-well chambered slides, which then were fixed with 2 % paraformaldehyde and permeabilized in methanol. After washing with PBS, slides were blocked with 2 % donkey serum for 0.5 h and then incubated with antibody against IRF1 (dilution 1:200) in 5 % donkey serum at 4 °C overnight. Negative controls were performed by omitting the primary antibody. Slides were rinsed and incubated with secondary antibodies at 37 °C for 1.0 h. Nuclei were counterstained with 4′,6-diamidino-2-phenylindole (DAPI) (1:1,500). Then the slides were immediate analyzed by a laser confocal scanning microscopy. Immunostaining density was quantified using Image J analysis.

### ChIP-qPCR analysis

Chromatin immunoprecipitation (ChIP) was carried out according to the instructions of the EZ-ChIP™ Chromatin immunoprecipitation kit (Millipore). After ChIP, the DNA precipitated by the anti-IRF1 antibody was detected with q-PCR, which was conducted in a final volume of 25 μl containing 12.5 μl of 2 × SYBR Mix, Taq DNA Polymerase (BioEasy, Hangzhou, China), 1 μl each of forward primer and reverse primers (10 μM), and 6 μl of DNA template under the following conditions: the template was first denatured at 94 °C for 10 min, then subjected to 50 cycles of amplification (94 °C for 20 s, 60 °C for 1 min), 95 °C for 2 min, 72 °C for 1 min, 95 °C for 30 s, and 55 °C for 10 s (repeat 80 times), 30 °C for 1 min. After PCR, relative data quantification was performed using the 2^−ΔΔCt^ method, and the result was calculated in the form of %Input, which was given by the following formula: %Input = 2^(Ctinput−CtChIP)^ × input dilution factor × 100. A segment of the HOTAIR promoter containing the IRF1-binding sites was amplified using the primers 5′-GCCCTGATTCTCTGGCTTT-3′ (forward) and 5′-CTGGAACAGATCCCAAACA-3′ (reverse).

### siRNAs and transfections

The DNA sequences (forward 5′- GCACCAGTGATCTGTACAA-3′, reverse 5′- CCAGATCCCATGGAAGCAT-3′) corresponding to siRNAs was used to target IRF1. Plasmids expressing siRNAs were constructed by inserting the coding sequences into the pcDNA3.1 vector (Invitrogen, USA). The cells were transfected using Lipofectamine 2000 in Opti-Mem according to the manufacturer’s protocol. The medium was replaced 8 h later, and the cells were collected for the subsequent experiments 48 h post-transfection.

### Statistical analysis

The results are presented as mean ± standard deviation (SD), for at least three-independent experiments. Tumor incidences were compared using the *χ*2 test. Other data were analyzed by one-way ANOVA followed by Tukey’s test for multiple comparisons. Significance of difference was set at *P* < 0.05.

## Results

### Dp effectively suppresses carcinogen-induced chronic cellular breast carcinogenesis

**Fig. 1:**
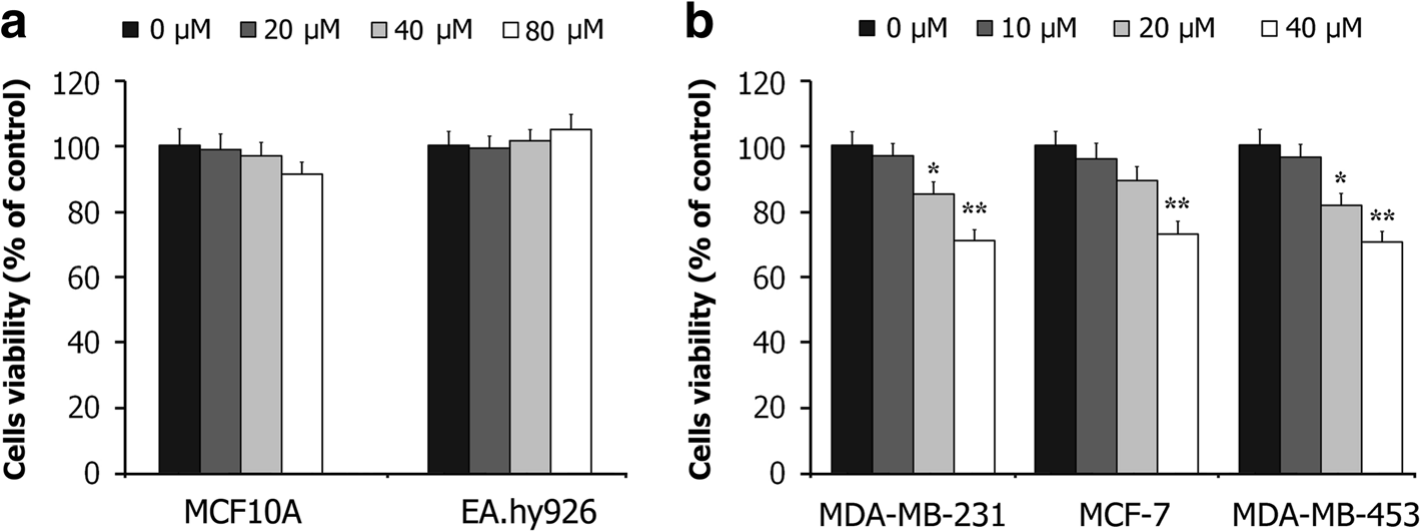
The effects of Dp treatment for 48 h on the cells viability in human breast epithelial cell line MCF10A, human vascular endothelial cell line EA.hy926 (**a**), and in breast cancer cell lines MDA-MB-231, MCF-7 and MDA-MB-453 (**b**). The data are presented as the means ± SD (*n* = 3). ^*^ *P* < 0.05 and ^**^ *P* < 0.01 compared with the control

First, we assessed the effects of Dp on the cells viability in human breast epithelial cell line MCF10A and in human vascular endothelial cell line EA.hy926. As shown in Fig. 1a, Dp has no apparent cytotoxic effects on these cell lines. The assays also indicated that Dp treatment significantly reduces cells viability in breast cancer cells (Fig. 1b).

**Fig. 2:**
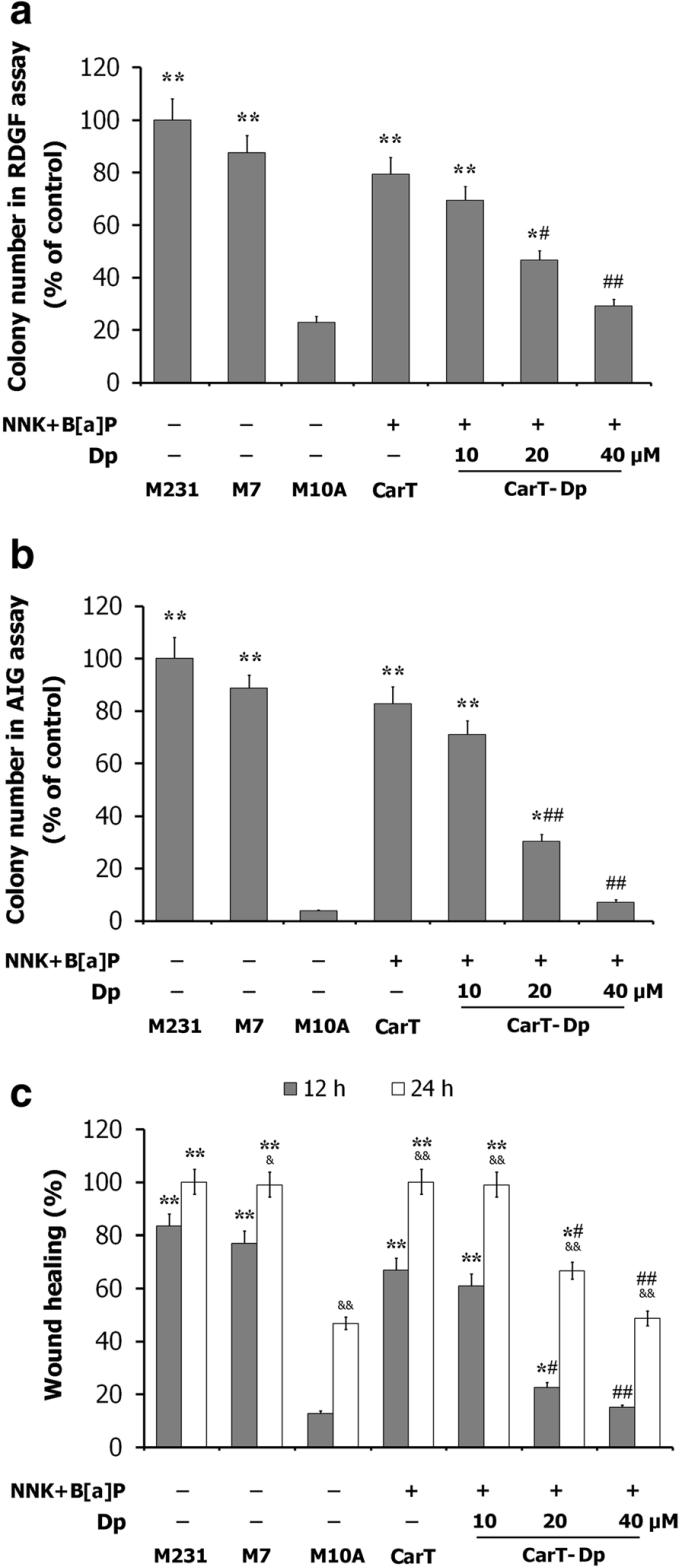
The effects of Dp on the carcinogens-induced chronic cellular breast carcinogenesis. MCF10A cells were treated with carcinogens NNK and B[a]P or co-treated with carcinogens and Dp (10, 20 and 40 μM respectively) for 30 days. Then cancer-associated properties were evaluated by RDGF assay (**a**), AIG assay (**b**) and scratch/wound healing assay (**c**) in MDA-MB-231 (M231), MCF-7 (M7), MCF10A (M10A), 30-d carcinogens-treated MCF10A cells (CarT), and 30-d carcinogens plus Dp co-treated MCF10A cells (CarT-Dp). The data are presented as the means ± SD (*n* = 3). ^*^ *P* < 0.05 and ^**^ *P* < 0.01 compared with MCF10A cells; ^**#**^ *P* < 0.05 and ^**##**^ *P* < 0.01 compared with CarT cells; &*P* < 0.05; &&*P* < 0.01 compared with cells in 12 h

MCF10A cells were treated with carcinogens or co-treated with carcinogens plus Dp for 30 days. Then, the cancer-associated properties of the treated cells were evaluated. As shown in Fig. 2a and b, carcinogens-treated cells (CarT) showed aberrantly increased cell survival adapted to RDGF and AIG, indicating cellular carcinogenic transformation. Compared with CarT, cells co-treated with carcinogens plus Dp (CarT-Dp) exhibited a significantly lower acquisition of RDGF and AIG. Similarly, the wound-healing assay also showed that CarT cells increased proliferation and mobility to heal the wound, which could be effectively suppressed by Dp co-treatment (Fig. 2c).

### Dp down-regulates the expression of HOTAIR in breast carcinogenesis and breast cancer cells in vitro and in vivo

**Fig. 3:**
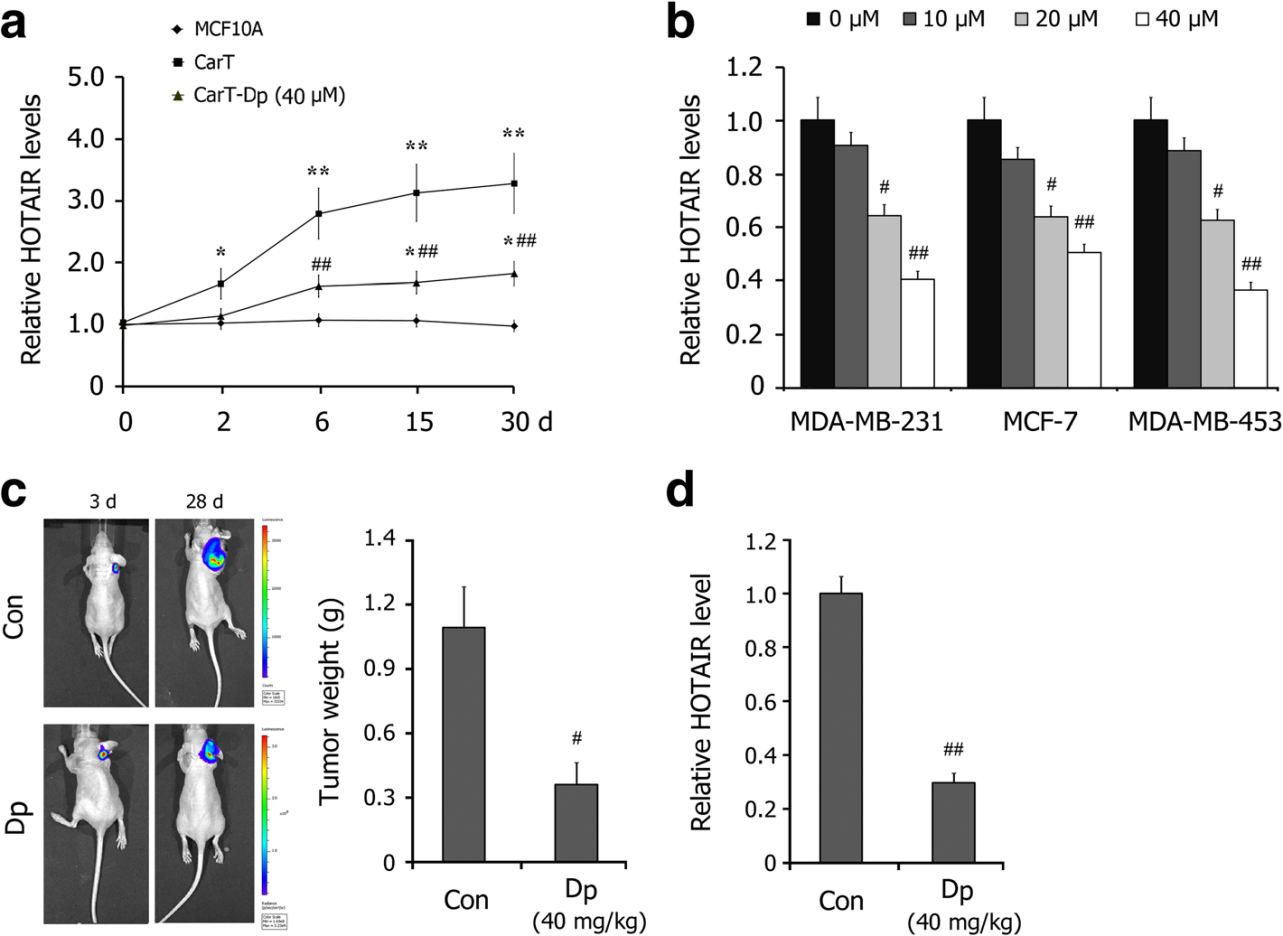
Effects of Dp treatment on the expressions of HOTAIR in breast carcinogenesis and in breast cancer cells. **a** The levels of HOTAIR in MCF10A, CarT and CarT-Dp (40 μM Dp) cells in the cellular breast carcinogenesis model were detected by qRT-PCR. **b** Effects of Dp treatment for 24 h on the levels of HOTAIR in breast cancer cells MDA-MB-231, MCF-7 and MDA-MB-453. **c** The effect of Dp administration (40 mg/kg) on the xenografted breast tumors of MDA-MB-231-Luc-GFP cells monitored by in vivo luminescence imaging system. The tumors weight was measured at day 28 post-implantation. **d** The effect of Dp administration (40 mg/kg) on the level of HOTAIR in xenografted breast tumors. The data are presented as the mean ± SD (*n* = 3). ^*^ *P* < 0.05, ^**^ *P* < 0.01 compared with MCF10A cells; ^#^ *P* < 0.05, ^##^ *P* < 0.01 compared with CarT cells or the control

We used qRT-PCR to detect the changes in the HOTAIR levels in cellular breast carcinogenesis, and the data revealed a significant up-regulation of HOTAIR in CarT cells in a time-dependent manner, which was significantly decreased by Dp co-treatment (Fig. 3a). Meanwhile, we also determined the effects of Dp on the HOTAIR levels in breast cancer cells. The results showed that Dp treatment effectively repressed the expression of HOTAIR in MDA-MB-231, MCF-7 and MDA-MB-453 cells (Fig. 3b).

Furthermore, we detected the effect of Dp on HOTAIR expression in vivo. A luciferase-expressing breast cancer cell line, MDA-MB-231-Luc-GFP, was injected into the mammary fat pad of female BALB/c mice. Stable expression of firefly luciferase and an in vivo luminescence imaging system (IVIS) allows for the monitoring of tumor growth. At day 3 post-implantation, mice with similar tumor loads were randomized and separated into two treatment groups. After an additional 25 days of treatment, the tumors were isolated from the mice. As shown in Fig. 3c, Dp administration (40 mg/kg) reduced the intensity and size of the in vivo luminescence in the animals, effectively inhibiting the growth of tumors. qRT-PCR detections showed that Dp administration significantly decreased the level of HOTAIR in xenografted breast tumors in athymic mice (Fig. 3d). These data indicate that Dp treatment significantly down-regulates HOTAIR expression in breast carcinogenesis and breast cancer cells in vitro and in vivo.

### Dp down-regulates HOTAIR by inhibiting Akt activation and promoting IRF1

**Fig. 4:**
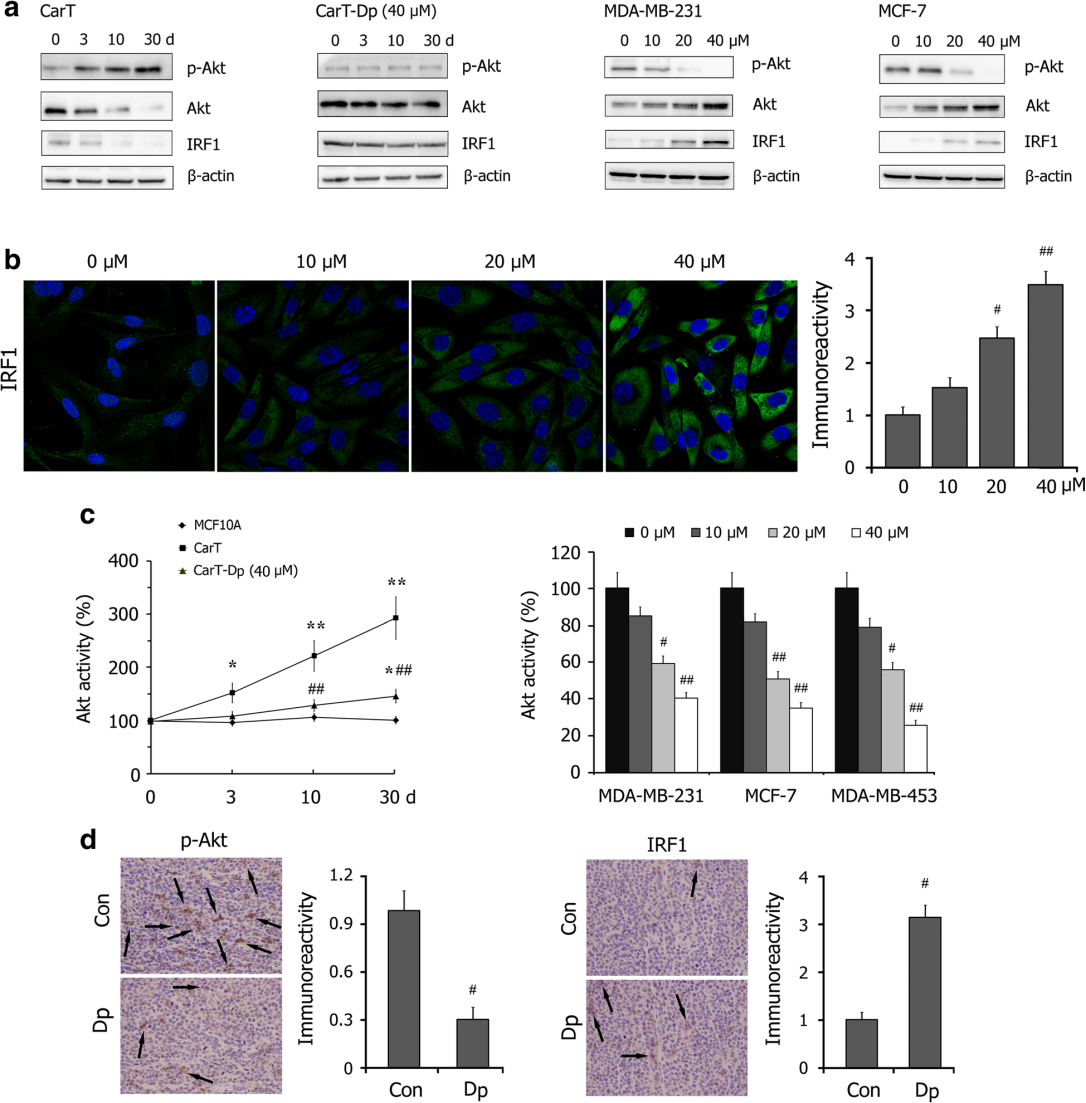
Effects of Dp treatment on Akt/IRF1 signaling pathway in breast carcinogenesis and breast cancer cells. **a** Western blot detections for the levels of p-Akt, Akt and IRF1 in CarT and CarT-Dp (40 μM Dp) cells in the cellular breast carcinogenesis model, as well as in breast cancer cells MDA-MB-231 and MCF-7 treated with Dp for 24 h. **b** Immunofluorescence analysis of IRF1 levels in breast cancer cells MDA-MB-231 treated with different concentrations of Dp for 24 h. Immunostaining density was quantified using Image J analysis. **c** The levels of Akt activity in MCF10A, CarT and CarT-Dp (40 μM Dp) cells in the cellular breast carcinogenesis model, as well as in breast cancer cells treated with Dp for 24 h. **d** Immunohistochemistry detections of p-Akt and IRF1 expression in breast tumors in athymic mice. Immunostaining density was quantified using Image J analysis. The data are presented as the means ± SD (*n* = 3). ^*^ *P* < 0.05 and ^**^ *P* < 0.01 compared with MCF10A cells; ^**#**^ *P* < 0.05 and ^**##**^ *P* < 0.01 compared with CarT cells or the control

Because of the important role of Akt/IRF1 signaling in the regulation of HOTAIR expression, we evaluated the effect of Dp on Akt activation and the IRF1 levels. As shown in Fig. 4a, western blot assays showed that the level of p-Akt and the ratio of p-Akt/Akt in CarT cells significantly increased in a time-dependent manner, indicating significant Akt activation in carcinogenesis. Co-treatment with Dp effectively inhibited Akt activation and the down-regulation of IRF1 in breast carcinogenesis. Moreover, the results indicated that Dp significantly inhibits Akt activation and up-regulates IRF1 in a dose-dependent manner in breast cancer cells.

To verify these findings, we measured the activity of Akt with an ELISA-based kinase activity assay. Consistent with the western blot assays, the data confirmed that Dp treatment effectively inhibited Akt activity in breast carcinogenesis and breast cancer cells (Fig. 4c). Furthermore, immunohistochemistry detections confirmed that Dp administration decreased the level of p-Akt and promoted IRF1 in xenografted breast tumors in vivo (Fig. 4d).

### Akt/IRF1/HOTAIR signaling plays a crucial role in Dp-induced cytotoxicity of breast cancer cells

**Fig. 5:**
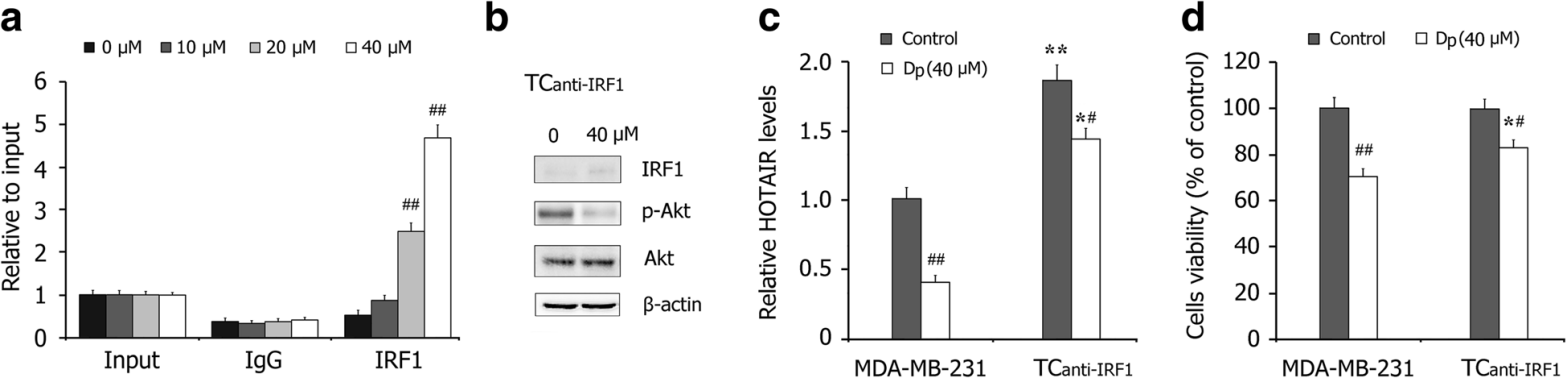
The role of Akt/HOTAIR signaling in Dp-induced cytotoxicity on breast cancer cells. **a** The ChIP-qPCR detections of IRF1 binding to the HOTAIR promoter in breast cancer MDA-MB-231 cells treated with Dp for 24 h. **b** Western blot detections of IRF1, p-Akt and Ake in Dp-treated MDA-MB-231 cells transfected with IRF1 siRNA (TC_anti-IRF1_). **c** Effects of Dp treatment for 24 h on the levels of HOTAIR in MDA-MB-231 cells and TC_anti-IRF1_ cells. **d** Effects of Dp treatment for 48 h on the cells viability in MDA-MB-231 cells and TC_anti-IRF1_ cells. The data are presented as the mean ± SD (*n* = 3). ^*^ *P* < 0.05 and ^**^ *P* < 0.01 compared with MDA-MB-231 cells; ^**#**^ *P* < 0.05 and ^**##**^ *P* < 0.01 compared with the control

We first performed ChIP-qPCR analysis and the results showed that Dp treatment effectively increases IRF1 binding to the HOTAIR promoter in MDA-MB-231 cells (Fig. 5a). We then blocked the Dp-induced promotion of IRF1 by transfecting cells with IRF1 siRNAs (TC_anti-IRF1_). As shown in Fig. 4b, IRF1 siRNAs blocked the effect of Dp on IRF1 expression in MDA-MB-231 cells. The qRT-PCR assays showed that the level of HOTAIR in TC_anti-IRF1_ cells was significantly increased and that the suppressive effect of Dp on HOTAIR was significantly reduced (Fig. 5c). The cell viability assay further revealed that the up-regulation of HOTAIR significantly decreased the cytotoxic effects of Dp on breast cancer cells (Fig. 5d). These findings indicate that the Akt/IRF1/HOTAIR signaling pathway plays a crucial role in the anti-cancer mechanism of Dp.

## Discussion

Approximately one-third of cancers in Western countries can be prevented by eating a plant food-based healthy diet and maintaining a physically active life style. Epidemiologic studies and meta-analysis confirmed that high consumption of fruits and vegetables is associated with a significantly reduced risk of breast cancer [19, 20]. Dietary flavonoids, a large group of polyphenolic compounds in fruits and vegetables, have been identified as potential chemopreventive components in the diet. Flavonoids are categorized into six major subclasses based on their range and structural complexity as follows: flavonols, flavones, flavan-3-ols, flavanones, anthocyanins and isoflavones. Anthocyanidines are abundant in colored berries, black currants, grapes, cabbages and other pigmented fruits and vegetables in the Western diet [21, 22]. The present study indicated that Dp, a major anthocyanin, effectively suppresses chemical carcinogen-induced chronic breast carcinogenesis. These findings provide useful insight regarding the role of diet in breast cancer prevention.

Protein-coding genes comprise only a small part of the genome, suggesting that non-coding RNAs (ncRNAs) may play a critical role in the regulation of cellular processes, such as cell growth, differentiation and apoptosis. ncRNAs are found throughout the genome [23, 24]. They can be divided into two major classes based on transcript size, small ncRNAs and long ncRNAs. The functions and clinical significance of short ncRNAs, such as miRNAs and siRNAs, have been extensively investigated and elucidated; however, lncRNAs were identified more recently, and their functions remain relatively unknown. The majority of lncRNAs functions with DNA-binding proteins, such as chromatin modifying complexes, and play roles in the epigenetic regulation of multiple genes [25–27].

The HOTAIR gene is located within the HOXC gene cluster on chromosome 12 and encodes a 2.2-kb lncRNA. Studies showed that HOTAIR is aberrantly up-regulated in many cancers, including breast cancer, colorectal cancer, and prostate cancer. HOTAIR can interact with the polycomb repressive complex 2 (PRC2) and lysine specific demethylase 1 (LSD1) complexes, resulting in the epigenetic silencing of many related genes [16, 28]. Several studies indicated that the expression of HOTAIR frequently changes during malignant transformation and may be a key molecule in breast carcinogenesis and cancer progression, with the potential to serve as a novel biomarker and therapeutic target. Our study showed that the expression of HOTAIR was significantly increased in breast carcinogenesis and that Dp co-treatment effectively inhibited the aberrant regulation of HOTAIR. Furthermore, Dp significantly down-regulated HOTAIR expression in breast cancer cells. These findings indicate that the suppression of HOTAIR may be an important mechanism of Dp-induced anti-cancer effects. To explore the mechanism by which Dp down-regulates HOTAIR expression, we investigated the effects of Dp on Akt activation in breast carcinogenesis and breast cancer cells. The data revealed that Dp treatment effectively inhibits Akt activity and consequently promotes IRF1 expression, which decreases HOTAIR expression. Further studies confirmed that blocking the Dp-induced suppression of HOTAIR significantly decreased the anti-cancer effects of Dp on breast cancer cells.

## Conclusion

Our study showed the effective chemopreventive effects of Dp on chemical carcinogen-induced breast carcinogenesis, and we found that Dp down-regulated HOTAIR expression by suppressing Akt activation in breast carcinogenesis and breast cancer cells.

## Abbreviations

AIG, anchorage-independent cell growth; B[a]P, benzo[a]pyrene; BC, breast cancer; CarT, carcinogens-treated cells; CarT-Dp, cells co-treated with carcinogens plus Dp; CM, complete medium; DMSO, dimethylsulfoxide; Dp, delphinidin-3-glucoside; HOTAIR, HOX transcript antisense RNA; IRF1, interferon regulatory factor-1; IVIS, in vivo luminescence imaging system; lncRNA, long non-coding RNA; MTT, 3-(4,5-dimethylthiazol-2-yl)-2,5-diphenyl tetrazolium bromide; ncRNAs, non-coding RNAs; NNK, 4-(methylnitrosamino)-1-(3-pyridyl)-1-butanone; PBS, phosphate buffered saline; RDGF, reduced dependence on growth factors; TCanti-IRF1, cells with IRF1 siRNAs
